# Monosynaptic Retrograde Tracing From Prelimbic Neuron Subpopulations Projecting to Either Nucleus Accumbens Core or Rostromedial Tegmental Nucleus

**DOI:** 10.3389/fncir.2021.639733

**Published:** 2021-02-25

**Authors:** Adelis M. Cruz, Tabitha H. Kim, Rachel J. Smith

**Affiliations:** ^1^Department of Psychological and Brain Sciences, Texas A&M University, College Station, TX, United States; ^2^Institute for Neuroscience, Texas A&M University, College Station, TX, United States

**Keywords:** rabies, addiction, reinstatement, RMTg, medial prefrontal cortex (mPFC)

## Abstract

The prelimbic (PL) region of the medial prefrontal cortex (mPFC) has been implicated in both driving and suppressing motivated behaviors, including cocaine-seeking in rats. These seemingly opposing functions may be mediated by different efferent targets of PL projections, such as the nucleus accumbens (NAc) core and rostromedial tegmental nucleus (RMTg), which have contrasting roles in reward-seeking behaviors. We sought to characterize the anatomical connectivity differences between PL neurons projecting to NAc core and RMTg. We used conventional retrograde tracers to reveal distinct subpopulations of PL neurons projecting to NAc core vs. RMTg in rats, with very little overlap. To examine potential differences in input specificity for these two PL subpopulations, we then used Cre-dependent rabies virus (EnvA-RV-EGFP) as a monosynaptic retrograde tracer and targeted specific PL neurons *via* injections of retrograde CAV2-Cre in either NAc core or RMTg. We observed a similar catalog of cortical, thalamic, and limbic afferents for both NAc- and RMTg-projecting populations, with the primary source of afferent information arising from neighboring prefrontal neurons in ipsilateral PL and infralimbic cortex (IL). However, when the two subpopulations were directly compared, we found that RMTg-projecting PL neurons received a greater proportion of input from ipsilateral PL and IL, whereas NAc-projecting PL neurons received a greater proportion of input from most other cortical areas, mediodorsal thalamic nucleus, and several other subcortical areas. NAc-projecting PL neurons also received a greater proportion of contralateral cortical input. Our findings reveal that PL subpopulations differ not only in their efferent target but also in the input specificity from afferent structures. These differences in connectivity are likely to be critical to functional differences of PL subpopulations.

## Introduction

The prelimbic (PL) region of the medial prefrontal cortex (mPFC) plays roles in both driving and suppressing motivated behaviors, including drug-seeking and conditioned fear (Moorman et al., 2015; Gourley and Taylor, 2016). Opposing behavioral functions for PL may be mediated by distinct efferent projections. This is supported by previous work demonstrating bidirectional behavioral effects after optogenetic stimulation of distinct PL projection pathways, including PL projections to lateral habenula vs. dorsal raphe in a forced swim task (Warden et al., 2012), nucleus accumbens (NAc) vs. basolateral amygdala (BLA) in an active avoidance task (Diehl et al., 2020), and NAc vs. paraventricular nucleus of the thalamus during cue-induced reward-seeking (Otis et al., 2017). Additionally, whereas PL projections to NAc core have been shown to drive cue-induced reinstatement of cocaine-seeking (McFarland et al., 2003; Stefanik et al., 2013, 2016; McGlinchey et al., 2016; James et al., 2018), we recently showed that PL projections to the rostromedial tegmental nucleus (RMTg) play a suppressive role in cue-induced reinstatement (Cruz et al., 2020).

Here, we sought to characterize anatomical connectivity differences between PL neurons projecting to NAc core and RMTg, given that these two efferent targets often have opposing influences on reward-seeking behavior. The NAc core is critically involved in driving motivated behavior and action initiation, particularly when guided by incentive stimuli (Du Hoffmann and Nicola, 2014; Floresco, 2015; Hamid et al., 2016; Syed et al., 2016; Mohebi et al., 2019; Sicre et al., 2019). Conversely, the RMTg, or tail of the ventral tegmental area (tVTA), is involved in behavioral inhibition and aversive valence encoding, *via* its inhibitory influence on dopamine neurons in the ventral tegmental area (Jhou et al., 2009a,b, 2013; Kaufling et al., 2009, 2010; Balcita-Pedicino et al., 2011; Barrot et al., 2012; Bourdy et al., 2014; Vento et al., 2017; Li et al., 2019; Smith et al., 2019). Previous work has demonstrated that mPFC projections to a variety of brain structures typically arise from different subpopulations of mPFC neurons (Akintunde and Buxton, 1992; Pinto and Sesack, 2000; Gabbott et al., 2005). Therefore, we hypothesized that distinct subpopulations of PL neurons project to NAc core vs. RMTg, and we sought to investigate connectivity differences for these subpopulations, as differences in input/output specificity are likely to be critical to their functional differences.

**Figure 1:**
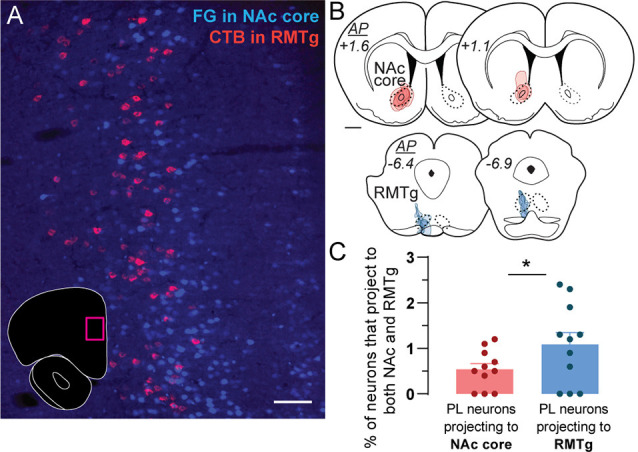
Retrograde tracing of prelimbic (PL) neurons projecting to nucleus accumbens (NAc) core and rostromedial tegmental nucleus (RMTg). **(A)** Representative photo of retrogradely-traced neurons in PL following injections of Fluoro-Gold (FG) in NAc core (blue) and CTB in RMTg (magenta). Photo location is shown in the inset (magenta box). Scale bar = 100 μm. **(B)** Location and spread for retrograde tracer injections in NAc core (FG, red) and RMTg (CTB, blue). Each rat is represented by one translucent outline, and outlines are overlapped. Anterior-posterior (AP) levels represent mm from bregma. Scale bar = 1 mm. **(C)** Percentage of PL neurons that project to both NAc core and RMTg (i.e., double-labeled for both CTB and FG), as a proportion of the total number of PL neurons projecting to NAc core (all FG-labeled cells) or RMTg (all CTB-labeled cells). Averages (± SEM) are shown, as well as individual data points for each rat (*n* = 11, *p < 0.05).

Using conventional retrograde tracers, we show that PL neurons projecting to NAc core and RMTg are markedly separate, forming two largely distinct sublayers of pyramidal cells in rats (Figure 1). Therefore, we investigated these two PL projection pathways in terms of afferent connectivity, considering that input specificity might influence differential recruitment of these populations during behavior. We used Cre-dependent rabies (EnvA-RV-EGFP) as a monosynaptic retrograde tracer to determine direct afferents to the two different PL projection neuron subpopulations (Wickersham et al., 2007a,b, 2010; Callaway, 2008; Wall et al., 2010, 2013; Watabe-Uchida et al., 2012). Cre was expressed specifically in RMTg- or NAc-projecting neurons using retrograde CAV2-Cre microinjected into the efferent target (RMTg or NAc), followed by microinjection of AAV helper viruses and EnvA-RV-EGFP into PL. Our results revealed that PL neurons projecting to either NAc core or RMTg receive input from similar afferents, but differ in the proportions of input arising from these afferents.

## Materials and Methods

### Animals

Male Sprague Dawley rats (initial weight 250–300 g; Charles River, Raleigh, NC, USA) were single-housed under a 12-h reverse light/dark cycle (ZT0 = 19:00) and had access to food and water *ad libitum*. Animals were housed in a temperature- and humidity-controlled animal facility with AAALAC accreditation. All experiments were approved by the Institutional Animal Care and Use Committee at Texas A&M University and conducted according to specifications of the National Institutes of Health as outlined in the Guide for the Care and Use of Laboratory Animals.

### Viruses

The transduction and monosynaptic spread of glycoprotein (G)-deleted rabies virus (RV) with EnvA pseudotyping (EnvA-RV-EGFP) is limited to neurons expressing TVA receptor and rabies glycoprotein (RG) in a Cre-dependent manner (Wickersham et al., 2007a, b; Watabe-Uchida et al., 2012). We targeted specific PL neurons by injecting either NAc core or RMTg with retrograde Cre-expressing virus (CAV2-Cre) and injecting PL with Cre-dependent helper AAV viruses (TVA and RG) and EnvA-RV-EGFP.

EnvA-RV-EGFP (titer of 4.3 × 10e8) was provided by Ed Callaway *via* the Gene Transfer, Targeting, and Therapeutics Facility at the Salk Institute (Wickersham et al., 2007a). AAV-TVA (AAV1-EF1a-FLEX-TVAmCherry, a titer of 4 × 10e12) and AAV-RG (AAV1-CA-FLEX-RG, a titer of 4 × 10e12) were obtained through the viral vector core at the University of North Carolina (developed by Watabe-Uchida et al., 2012). CAV2-Cre (CAV2-CMV-Cre recombinase) was provided by Eric J. Kremer at the Institut de Genetique Moleculaire de Montpellier (Kremer et al., 2000). We diluted CAV2-Cre 1:10 in 10% glycerol in PBS for a final titer of 10.3 × 10e11 ppml, based on preliminary experiments indicating cell toxicity at higher titers. All viral procedures were approved by the Institutional Biosafety Committee at Texas A&M University and conducted according to specifications of the National Institutes of Health.

### Stereotaxic Surgery

Rats were anesthetized with vaporized isoflurane (induced at 5%, maintained at 1–2%), given the analgesic ketoprofen (2 mg/kg, s.c.), and placed in a stereotaxic frame (Kopf, Tujunga, CA, USA). A skin incision was made over the skull and holes were drilled in the skull over the target sites. Intracranial injections were made using a pulled glass micropipette and Nanoject 2010 injector (World Precision Instruments, Sarasota, FL, USA). After the injection, the pipette was slowly raised from the brain and the skin was stapled closed.

For conventional retrograde tracing, rats were given unilateral microinjections of 100 nl of 2% Fluoro-Gold (FG, Fluorochrome, Denver, CO, USA) into unilateral NAc core (AP +1.8, ML +2.6, DV –7.3 from bregma, 6°) and 100 nl of 0.2% cholera toxin subunit b (CTB, List Biological Laboratories, Campbell, CA, USA) into RMTg (AP –7.2, ML +1.9, DV –7.6 from dura, 10°). FG was used only in NAc because it tended to cause toxicity in RMTg, evident by circling of animals after unilateral injection. Rats were sacrificed at least 1 week after injections.

For monosynaptic retrograde tracing, rats were given two surgeries. During the first stereotaxic surgery, CAV2-Cre (1,000 nl) plus biotin dextran (10 K MW, 0.2% final volume, for visualization of injection site) was injected unilaterally into either NAc core (AP +1.8, ML +2.6, DV –7.3 from bregma, 6°) or RMTg (AP –7.2, ML +1.8, DV –7.6 from dura, 10°), and a mixture of AAV-RG and AAV-TVA (1,000 nl of a 1:1 mix) was injected into ipsilateral PL (AP +3.1, ML +1.2, DV –3.8 from skull, 12°). Three weeks later during a second stereotaxic surgery, EnvA-RV-EGFP (1,000 nl) was injected into the same location in ipsilateral PL. Rats were sacrificed 7 days after rabies injections.

### Tissue Processing

Rats were deeply anesthetized with isoflurane and then transcardially perfused with 0.9% NaCl followed by 10% neutral-buffered formalin *via* a peristaltic pump (Cole Parmer, Vernon Hills, IL, USA). Brains were collected, post-fixed in 10% formalin overnight at 4°C, placed in 20% sucrose in phosphate-buffered saline (PBS) with 0.02% sodium-azide at 4°C for ≥2 days, frozen in dry ice, cut into 40-μm thick coronal sections on a cryostat, and collected into PBS-azide for storage before immunohistochemistry (IHC). For IHC, all incubations and rinses took place at room temperature on a shaker. Sections were rinsed in PBS three times between steps.

For FG and CTB tracing, free-floating sections were incubated overnight in goat anti-CTB (1:50 K, List Biological Labs Cat# 703, RRID: AB_10013220) in PBS with 0.25% Triton X-100 (PBST, Sigma–Aldrich), and then incubated for 1 h in Alexa Fluor 594-conjugated donkey anti-goat (1:500 in PBST, Jackson Immunoresearch Labs cat# 705-585-147, RRID:AB_2340433). Sections were mounted onto glass slides, coverslipped with ProLong Diamond Antifade (Thermo Fisher Scientific, Fair Lawn, NJ, USA), sealed with clear nail polish, and stored at 4°C.

For RV tracing, one well (every 12th section) was used for mCherry/EGFP fluorescence (for counting starter cells), and one well (every 12th section) was used for IHC to visualize mCherry/EGFP with DAB (for counting inputs to PL). For fluorescence, sections were mounted onto glass slides, coverslipped with ProLong Diamond Antifade, sealed with clear nail polish, and stored at 4°C. For DAB, free-floating sections were incubated for 15 min in 0.3% H_2_O_2_ in PBS, overnight in mouse anti-DsRed (1:2 K in PBST, Takara Bio Cat# 632392, RRID: AB_2801258), 1 h in biotinylated donkey anti-mouse (1:500 in PBST, Jackson Immunoresearch Labs Cat# 715–065–151, RRID: AB_2340785), and 45 min in ABC (Vector Elite Kit, 1:500 in PBST, Vector). The reaction was visualized *via* incubation for 10 min in 0.025% 3,3′-diaminobenzidine (DAB), 0.05% nickel ammonium sulfate, and 0.015% H_2_O_2_ (to yield black DAB color). Sections were then incubated overnight in rabbit anti-GFP (1:50 K in PBST, Abcam Cat# ab290, RRID:AB_303395), 1 h in biotinylated donkey anti-rabbit (1:500 in PBST, Jackson Immunoresearch Labs Cat# 711-065-152, RRID: AB_2340593), 45 min in ABC (Vector Elite Kit, 1:500 in PBST, Vector), and visualized *via* the same DAB reaction but without nickel (to yield brown DAB color). Sections were mounted onto Superfrost Plus slides, dried, counterstained with Methyl Green (0.5% in sodium acetate buffer, Sigma–Aldrich), and coverslipped with Permount (Thermo Fisher Scientific, Fair Lawn, NJ, USA).

### Data Analysis

**Figure 2:**
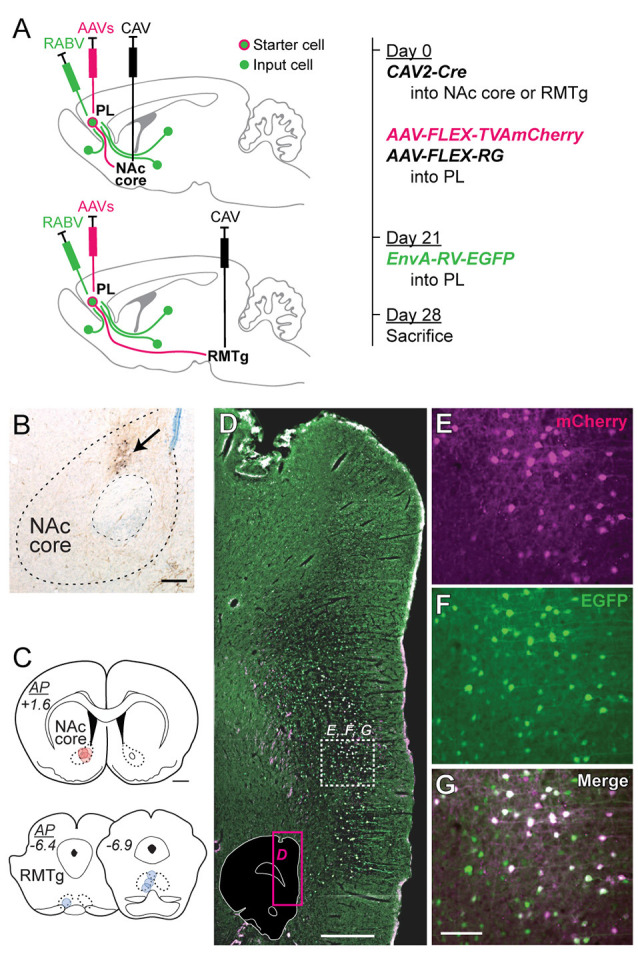
Virus injection strategy for labeling monosynaptic inputs to NAc core-projecting vs. RMTg-projecting PL neurons. **(A)** CAV2-Cre was injected into either NAc core (upper schematic) or RMTg (lower schematic), and rabies helper viruses (AAVs) were injected into PL during the initial surgery. EnvA-RV-EGFP was injected into PL 21 days later. Rats were sacrificed 7 days later. Brains were analyzed for starter cells (mCherry + EGFP) and input cells (EGFP). **(B)** Representative photo of CAV2-Cre injection site in NAc core with biotin dextran marking the center of the injection (arrow). Scale bar = 200 μm. **(C)** Location for CAV2-Cre injections in NAc core (red) or RMTg (blue). Each rat is represented by one translucent dot marking the center of the injection site. Scale bar = 1 mm. **(D)** Representative low-magnification fluorescent photo of EnvA-RV-EGFP labeling in PL at the injection site. Photo location is shown in the inset (magenta box). White dashed-outline box indicates the area shown at higher magnification in panels **(E–G)**. Scale bar = 500 μm. **(E)** mCherry expression (pseudocolored as magenta, from AAV helper viruses). **(F)** EGFP expression (green, from EnvA-RV-EGFP). **(G)** Merged image showing both mCherry and EGFP (with dual fluorescence shown in white). Neurons expressing both mCherry and EGFP are starter cells for retrograde labeling, whereas neurons expressing only EGFP are monosynaptic afferents to the starter cells. Scale bar = 100 μm.

Fluorescent and brightfield images were acquired using an Olympus BX51 microscope. For CTB and FG tracing, neurons were counted in PL on two sections per rat. Four rats were excluded from CTB/FG analysis due to small/missing RMTg injections. One rat was removed from CTB/FG analysis after being identified as a significant outlier (Grubb’s method) due to a higher percentage of double labeling. The remaining data were analyzed with a paired *t*-test. For rabies tracing, all EGFP-labeled neurons were counted on all sections stained with DAB (every 12th section) and categorized into brain regions according to boundaries identified by Paxinos and Watson (2007). RMTg boundaries were defined (Figures 1B, 2C) according to Smith et al. (2019). A neuron was only counted if a soma was clearly visible. Fluorescent sections were used to count all double-labeled neurons (EGFP + mCherry) on every 12th section. Expression of EGFP alone indicated input cells, and expression of both mCherry and EGFP indicated starter cells. Inputs were normalized within each animal and calculated as a percentage of total inputs per rat. Brain regions were only included in analysis and figures if normalized inputs were ≥0.2% in ≥2 rats. Data were analyzed using two-way ANOVAs (with repeated measures when appropriate) and Sidak *post hoc* analyses.

## Results

### Conventional Retrograde Tracing in PL Targets

Sparse double labeling was seen in PL (Figure 1A) when conventional retrograde tracers (FG and CTB) were injected into ipsilateral NAc core and RMTg (Figure 1B; *n* = 11). We observed NAc-projecting PL neurons in layers II/III and upper layer V, whereas RMTg-projecting neurons were located in deeper layer V (Figure 1A). Double-labeled neurons (projecting to both NAc core and RMTg) represented only 0.5% ± 0.1 of PL neurons projecting to NAc core and 1.1% ± 0.3 of PL neurons projecting to RMTg (Figure 1C; *t*_(10)_ = 3.004, *p* = 0.013). These data indicate that PL neurons projecting to NAc core vs. RMTg are predominantly separate subpopulations.

### Monosynaptic Retrograde Tracing in PL Subpopulations

To determine whether these PL subpopulations differ in terms of input specificity, we conducted monosynaptic retrograde tracing in each projection subpopulation *via* retrograde Cre delivery (Figure 2A). CAV2-Cre was injected into either NAc core (*n* = 4) or RMTg (*n* = 4; Figures 2B,C), and Cre-dependent helper AAVs driving expression of TVA-mCherry and RG were injected into PL. Three weeks later, EnvA-RV-EGFP was injected into PL. Rabies only infects and monosynaptically spreads from neurons expressing both TVA and RG.

**Figure 3:**
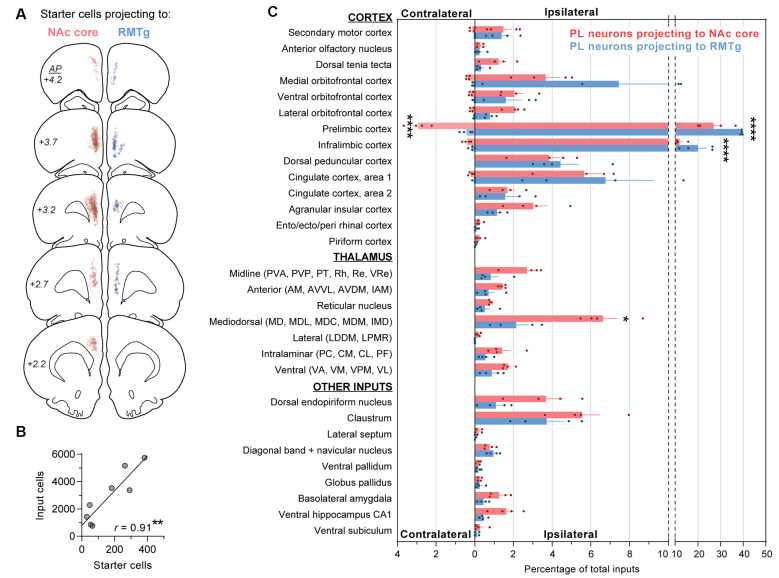
Quantification of monosynaptic inputs to NAc core-projecting vs. RMTg-projecting PL neurons. **(A)** Location of starter cells projecting to NAc core (red) or RMTg (blue). Each dot is one starter cell, and starter cells are shown together for all rats (*n* = 4 NAc core, 4 RMTg). **(B)** Relationship between number of starter cells and number of input cells (*r* = 0.91; **p < 0.01). **(C)** Contralateral and ipsilateral inputs to PL neurons projecting to NAc core vs. RMTg. Percentages reflect the number of cells quantified for each brain region divided by the total number of cells quantified for the whole brain per rat. Averages (±SEM) are shown only for brain regions with inputs ≥0.2% in ≥2 rats are shown. Statistically significant *post hoc* differences are shown (*p < 0.05; ****p < 0.0001). Abbreviations for thalamic nuclei: AM, anteromedial; AVDM, dorsomedial part of anteroventral; AVVL, ventrolateral part of anteroventral; CL, centrolateral; CM, central medial; IAM, interanteromedial; IMD, intermediodorsal; LDDM, dorsomedial part of lateroventral; LPMR, mediorostral part of lateral posterior; MD, mediodorsal; MDC, central part of mediodorsal; MDL, lateral part of mediodorsal; MDM, medial part of mediodorsal; PC, paracentral; PF, parafascicular; PT, paratenial; PVA, anterior part of paraventricular; PVP, posterior part of paraventricular; Re, reuniens; Rh, rhomboid; VA, ventral anterior; VL, ventrolateral; VM, ventromedial; VPM, ventral posteromedial; VRe, ventral reuniens.

**Figure 4:**
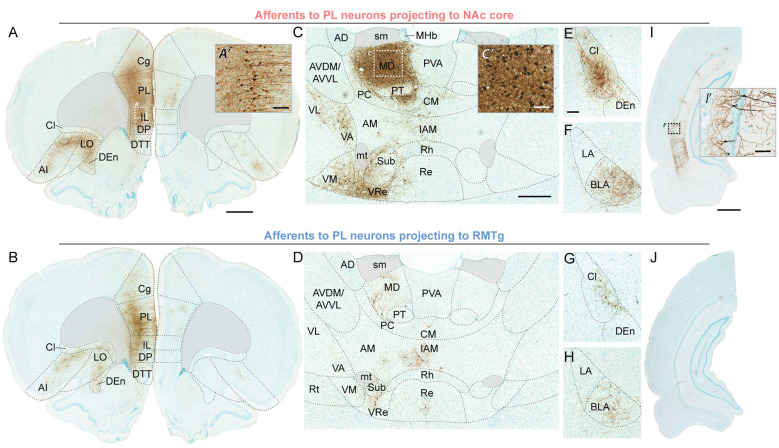
Representative brightfield photos showing monosynaptic afferents to PL neurons projecting to NAc core (top row) or RMTg (bottom row), with EGFP expression visualized *via* immunohistochemistry with DAB labeling (brown) and counterstaining *via* Methyl Green. **(A,B)** Ipsilateral and contralateral inputs in PFC; scale bar = 1 mm. High-magnification photo **(A’)** shows neuronal labeling in the area indicated by the white dashed-outline box; scale bar = 100 μm. **(C,D)** Inputs from thalamic nuclei; scale bar = 500 μm. High-magnification photo **(C’)** shows neuronal labeling in the area indicated by the white dashed-outline box; scale bar = 100 μm. **(E–H)** Inputs from claustrum, dorsal endopiriform nucleus, and BLA; scale bar = 200 μm. **(I,J)** Inputs from ventral hippocampus CA1; scale bar = 1 mm. High-magnification photo **(I’)** shows neuronal labeling in the area indicated by the black dashed-outline box; scale bar = 100 μm. AD, anterodorsal thalamic nucleus; AI, agranular insular cortex; AM, anteromedial thalamic nucleus; AVDM, dorsomedial part of anteroventral thalamic nucleus; AVVL, ventrolateral part of anteroventral thalamic nucleus; BLA, basolateral amygdala; Cg, cingulate cortex part 1; Cl, claustrum; CM, central medial thalamic nucleus; DEn, dorsal endopiriform nucleus; DP, dorsal peduncular cortex; DTT, dorsal tenia tecta; IAM, interanteromedial thalamic nucleus; IL, infralimbic cortex; LA, lateral amygdala; LO, lateral orbitofrontal cortex; MD, mediodorsal thalamic nucleus; MHb, medial habenular nucleus; mt, mammillothalamic tract; PC, paracentral thalamic nucleus; PL, prelimbic cortex; PT, paratenial thalamic nucleus; PVA, anterior part of paraventricular thalamic nucleus; Re, reuniens thalamic nucleus; Rh, rhomboid thalamic nucleus; Rt, reticular thalamic nucleus; sm, stria medullaris; Sub, submedius thalamic nucleus; VA, ventral anterior thalamic nucleus; VL, ventrolateral thalamic nucleus; VM, ventromedial thalamic nucleus; VRe, ventral reuniens thalamic nucleus.

We mapped and counted the number of starter cells (mCherry + EGFP) using fluorescent labeling (representative images in Figures 2D–G). We counted the number of input cells (EGFP) across different brain regions using immunohistochemistry with DAB labeling (representative images in Figure 4). Starter cells projecting to either NAc core or RMTg were primarily in PL, but also in neighboring infralimbic cortex (IL; Figure 3A). Although the average number of starter cells differed for NAc vs. RMTg (*t*_(6)_ = 5.50, *p* = 0.0015), the number of transsynaptically labeled cells was correlated with the number of starter cells (Figure 3B; *r* = 0.91, *p* = 0.0019).

Inputs were normalized within each animal and calculated as a percentage of total inputs per rat (Figure 3C). The total ipsilateral inputs were significantly greater than total contralateral inputs (main effect for contra/ipsi: *F*_(1,6)_ = 37,354, *p* < 0.0001), and the two PL subpopulations differed in terms of ipsilateral vs. contralateral inputs (main effect for PL subpopulation: *F*_(1,6)_ = 14.24, *p* = 0.009; interaction: *F*_(1,6)_ = 83.43, *p* < 0.0001). Therefore, we separately analyzed contralateral and ipsilateral inputs when comparing afferents for the two PL subpopulations. PL neurons projecting to NAc core vs. RMTg were significantly different in terms of ipsilateral input (two-way ANOVA, overall effect of PL subpopulation: *F*_(1,6)_ = 53.44, *p* = 0.0003; overall effect of afferents: *F*_(29,174)_ = 86.11, *p* < 0.0001; interaction: *F*_(29,174)_ = 5.05; *p* < 0.0001). We found significant differences for ipsilateral PL and IL (*p*’s < 0.0001), and mediodorsal thalamic nucleus (MD; *p* = 0.036). PL neurons projecting to NAc core vs. RMTg were also significantly different in terms of contralateral input (overall effect of PL subpopulation: *F*_(1,6)_ = 119.9, *p* < 0.0001; overall effect of afferents: *F*_(6,36)_ = 60.46, *p* < 0.0001; interaction: *F*_(6,36)_ = 34.06; *p* < 0.0001), with significant differences for contralateral PL (*p* < 0.0001).

For both populations, the primary source of afferent information was neighboring prefrontal neurons in PL and IL (Figures 4A,B). However, RMTg-projecting PL neurons received a significantly higher percentage of inputs from local ipsilateral PL and IL neurons (Figure 3C; PL: 39.4% ± 0.2 and IL: 20.1% ± 3.8), as compared to NAc-projecting PL neurons (PL: 26.9% ± 4.0 and IL: 11.8% ± 1.3). We also observed that RMTg-projecting PL neurons received a higher percentage of input from the medial orbitofrontal cortex (OFC), while NAc-projecting PL neurons received a higher percentage of input from the dorsal tenia tecta, lateral OFC, and agranular insular cortex. The percentage of inputs was similar for NAc-projecting and RMTg-projecting PL neurons for secondary motor cortex, anterior olfactory nucleus, ventral OFC, dorsal peduncular cortex, cingulate cortex areas 1 and 2, entorhinal/ectorhinal/perirhinal cortex, and piriform cortex. Interestingly, NAc-projecting PL neurons received a significantly greater percentage of contralateral cortical input from PL (2.92% ± 0.30) as compared to RMTg-projecting PL neurons (0.43% ± 0.15). NAc-projecting PL neurons also received a greater percentage of contralateral inputs from IL, secondary motor cortex, all areas of OFC (medial, ventral, and lateral), and cingulate cortex area 1.

Both PL subpopulations received prominent afferent information from the thalamus (Figures 4C,D), including subregions within the midline thalamic nuclei (anterior/posterior parts of paraventricular, paratenial, rhomboid, reuniens, ventral reuniens), anterior thalamic nuclei (anteromedial, ventrolateral/dorsomedial parts of anteroventral, interanteromedial), reticular thalamic nucleus, MD (mediodorsal, lateral/central/medial parts of mediodorsal, intermediodorsal), lateral thalamic nuclei (dorsomedial part of lateroventral, mediorostral part of lateral posterior), intralaminar thalamic nuclei (paracentral, central medial, centrolateral, and parafascicular), and ventral thalamic nuclei (ventral anterior, ventromedial, ventral posteromedial, ventrolateral; Figure 3C). NAc-projecting PL neurons received a higher percentage of inputs from all thalamic regions, as compared to RMTg-projecting PL neurons, with a significant difference observed for MD (6.6% ± 0.7 vs. 2.1% ± 0.6).

We also saw prominent inputs from the dorsal endopiriform nucleus, claustrum (Figures 4E,G), BLA (Figures 4F,H), and CA1 in the ventral hippocampus (Figures 4I,J). For all of these areas, NAc-projecting PL neurons received a greater percentage of input (Figure 3C). In contrast, the two subpopulations of PL neurons were similar in terms of afferent input arising from the lateral septum, diagonal band, and navicular nucleus, ventral pallidum, globus pallidus, and ventral subiculum (Figure 3C).

## Discussion

We found that PL neurons projecting to NAc vs. RMTg are distinct subpopulations (Figure 1). Pathway-specific monosynaptic retrograde tracing for PL neurons projecting to either NAc vs. RMTg (Figure 2) revealed differences in afferent information to the two PL subpopulations (Figures 3, 4). While RMTg-projecting PL neurons received a greater proportion of inputs from nearby sources in ipsilateral PL and IL, NAc-projecting PL neurons received a greater proportion of inputs from other cortical areas, thalamic nuclei, and subcortical areas. Also, NAc-projecting PL neurons received a greater proportion of contralateral cortical input. These data are the first to demonstrate anatomical connectivity differences between PL neurons projecting to NAc vs. RMTg, which may contribute to opposing functions of these pathways.

### PL Projection Subpopulations

We found very little overlap in PL neuron subpopulations projecting to NAc core vs. RMTg, with NAc-projecting PL neurons located in layers II/III and upper layer V, and RMTg-projecting neurons located in deeper layer V (Figure 1A). This corresponds with previous studies using conventional retrograde tracers (e.g., CTB) injected into various mPFC targets, which showed that distinct mPFC neuron subpopulations project to different subcortical targets, including NAc, BLA, MD, lateral hypothalamus, and numerous brainstem areas (Akintunde and Buxton, 1992; Pinto and Sesack, 2000; Gabbott et al., 2005). mPFC neurons in layers II/III, V, and VI have been shown to project to the striatum, while mPFC neurons in layer V project to multiple brainstem targets, including the parabrachial nucleus, periaqueductal gray, ventral tegmental area, dorsal raphe, the nucleus of the solitary tract, and ventrolateral medulla (Pinto and Sesack, 2000; Ding et al., 2001; Gabbott et al., 2005).

### Afferent Inputs to PL Subpopulations

Previous studies have mapped afferents to rat PL using conventional retrograde tracers (Condé et al., 1995; Hoover and Vertes, 2007). However, unlike conventional tracers, RV-EGFP allows the separation of starter cells from input cells, so that short-range projections within PL and IL can be assessed. Although previous studies have used RV-EGFP in mouse mPFC, they targeted all projection neurons (Ährlund-Richter et al., 2019), interneuron populations (Ährlund-Richter et al., 2019; Sun et al., 2019), or PL layer V (DeNardo et al., 2015). Here, we used RV-EGFP in rat mPFC and targeted PL neurons projecting to NAc core vs. RMTg to provide more detailed complexity about input specificity to PL subpopulations.

We observed prominent cortical inputs from mPFC (PL, IL, cingulate cortex, secondary motor cortex, dorsal peduncular cortex), OFC (with the highest percentage in medial), and agranular insular cortex, and limited labeling in the anterior olfactory nucleus, dorsal tenia tecta, piriform cortex, and ento/ecto/perirhinal cortex (Figures 3C, 4A,B). This parallels previous studies with conventional tracers (Condé et al., 1995; Hoover and Vertes, 2007). We found that for both NAc- and RMTg-projecting populations, the primary source of afferent information was neighboring prefrontal neurons in ipsilateral PL and IL, as shown previously with RV-EGFP targeting layer V neurons in PL (DeNardo et al., 2015). However, we also identified a major distinction between afferent inputs to NAc- and RMTg-projecting neurons. RMTg-projecting PL neurons received a higher percentage of input from ipsilateral PL and IL, as well as medial OFC, but a similar or lower percentage of input from all other cortical areas. In contrast, NAc-projecting PL neurons received a higher percentage of input from ipsilateral dorsal tenia tecta, lateral OFC, and agranular insular cortex. Additionally, NAc-projecting PL neurons received a greater percentage of contralateral input, including from PL, IL, cingulate cortex area 1, secondary motor cortex, OFC, and dorsal peduncular cortex. These findings indicate that RMTg-projecting neurons may be under greater local control, while NAc-projecting neurons have greater integration of information across hemispheres.

We found that NAc-projecting PL neurons received a higher proportion of inputs from all thalamic regions (Figures 3C, 4C,D). We observed the greatest thalamic labeling in MD and moderate labeling in midline thalamic nucleus, as per previous work (Condé et al., 1995; Hoover and Vertes, 2007). We also observed moderate labeling in intralaminar, ventral, anterior, and reticular nuclei, and some labeling in the lateral nucleus (but only in NAc-projecting PL neurons).

For areas outside the cortex and thalamus, we observed prominent labeling in dorsal endopiriform cortex and claustrum, and moderate labeling from ventral hippocampus CA1 and BLA (Figures 3C, 4E–J), as described previously (Condé et al., 1995; Hoover and Vertes, 2007; DeNardo et al., 2015). Interestingly, for all of these areas, NAc-projecting PL neurons received a greater percentage of input. Extensive previous work has characterized inputs to both pyramidal neurons and interneurons in PL arising from the ventral hippocampus (Ferino et al., 1987; Jay et al., 1989, 1992; Jay and Witter, 1991; Condé et al., 1995; Carr and Sesack, 1996; Gabbott et al., 2002; Hoover and Vertes, 2007) and BLA (Bacon et al., 1996; Gabbott et al., 2006; Dilgen et al., 2013; Ährlund-Richter et al., 2019). Additionally, we detected afferent information arising from the lateral septum, diagonal band and navicular nucleus, ventral pallidum, globus pallidus, and ventral subiculum. Although previous studies noted some input from lateral and posterior hypothalamus (Condé et al., 1995; Hoover and Vertes, 2007; DeNardo et al., 2015), we observed only sparse labeling (<0.2% in all rats) and thus these areas were excluded from analysis.

Notably, unlike previous studies using conventional retrograde tracers in PL (Condé et al., 1995; Hoover and Vertes, 2007), the current study using RV-EGFP as a tracer did not detect inputs from the monoaminergic nuclei of the brainstem, including ventral tegmental area, dorsal raphe, median raphe, and locus coeruleus, indicating that RV-EGFP does not spread transsynaptically to all retrograde afferent neurons. This is confirmed by previous studies showing a lack of brainstem inputs when using RV-EGFP in PL (DeNardo et al., 2015), and a paucity of dopamine inputs when using RV-EGFP in dorsal striatum as a monosynaptic tracer but not as a traditional retrograde tracer (Wall et al., 2013). These results indicate that the transsynaptic spread of RV-EGFP has reduced efficiency at monoaminergic synapses, possibly due to differences in synapse type, location, or distance, as compared to glutamatergic and GABAergic synapses, and this represents a significant limitation of transsynaptic tracing with RV-EGFP. Alternatively, these data may indicate that these PL subpopulations do not receive direct input from monoamine neurons. However, previous work has shown direct synaptic contact between NAc-projecting mPFC neurons and axon terminals containing tyrosine hydroxylase (Carr et al., 1999). Further studies are needed to determine whether RMTg-projecting PL neurons receive direct synaptic input from monoamine neurons.

### Technical Considerations

In this study, we used a two-helper-virus system, and this may have resulted in overestimating starter cells due to the assumption that each cell was successfully infected with both viruses. We did not demonstrate Cre dependence of the helper viruses in the current study (e.g., by including control animals lacking injection of CAV2-Cre), but previous work has shown limited expression of helper viruses in the absence of Cre (Watabe-Uchida et al., 2012; Wall et al., 2013; Schwarz et al., 2015). Additionally, it is important to note that our injections of CAV2-Cre likely were not restricted solely to RMTg, given the small size of RMTg as compared to NAc core and given that many injections show some spread outside the intended target (Figure 1B). It is important to note as well that these studies were performed in male rats; future studies are necessary to determine whether sex differences exist for these PL subpopulations. Finally, although RV-EGFP tracing reveals the presence of a connection between two neurons, it does not reveal information about the strength or function of the connection. One neuron may form many synapses onto many neurons and may provide a strong influence despite limited labeling with RV-EGFP. Therefore, additional studies are necessary to explore potential functional differences in afferent input to these PL subpopulations.

### Conclusions

The current work shows that PL subpopulations differ not only in their efferent target but also in the proportion of inputs they receive from a variety of afferent structures. When directly compared, RMTg-projecting PL neurons receive a greater proportion of input from nearby cortical input (ipsilateral PL and IL), whereas NAc-projecting PL neurons receive a greater proportion of input from other mPFC and cortical areas, thalamic nuclei, amygdala, and ventral hippocampus, as well as contralateral mPFC. These differences in afferent information may be critical to potential functional differences of these PL subpopulations and may be related to the contrasting roles played by NAc core and RMTg during reward-related behavior, including cocaine seeking. More generally, our data indicate that projection neuron subpopulation may be an important organizational feature of mPFC that has been mostly overlooked, and that differences in afferents, efferents, and function may be critical to the diverse functionality of mPFC across a range of behavioral responses.

## Data Availability Statement

The raw data supporting the conclusions of this article will be made available by the authors, without undue reservation.

## Ethics Statement

The animal study was reviewed and approved by the Institutional Animal Care and Use Committee at Texas A&M University.

## Author Contributions

RS designed the research. AC, TK, and RS performed the research. AC and RS analyzed the data and wrote the article. All authors contributed to the article and approved the submitted version.

## Conflict of Interest

The authors declare that the research was conducted in the absence of any commercial or financial relationships that could be construed as a potential conflict of interest.
